# Tolerability, Safety, and Pharmacokinetics of Ivermectin After Nasal Application in Healthy Adult Subjects

**DOI:** 10.1002/jcph.70137

**Published:** 2025-12-17

**Authors:** Stefan Wissel, Philipp Wissel, Matthias Rischer, Felix Häberlein, Hilde Riethmüller‐Winzen, Hanns Häberlein

**Affiliations:** ^1^ HWI Pharma Services GmbH Rülzheim Germany; ^2^ Dr. Riethmüller M/R/S GmbH Frankfurt am Main Germany; ^3^ Institute of Biochemistry and Molecular Biology Medical Faculty University of Bonn Bonn Germany

**Keywords:** ivermectin microsuspension, nasal application, nasal spray, pharmacokinetics, safety, tolerability

## Abstract

Nasal epithelium is the site of infection for SARS‐CoV2 viruses, with interactions of the viral spike protein with the ACE2 receptor of the host cell. Molecular docking studies have shown that ivermectin shields the spike protein and thereby prevents binding to ACE2. Nasal application of high doses of ivermectin could be the right therapeutic approach in the treatment and prevention of COVID‐19. Tolerability, safety, and pharmacokinetics of ivermectin, administered nasally as 5% microsuspension (F004), were investigated in a randomized, double‐blind, parallel‐groups, placebo‐controlled phase 1 study in 28 healthy adults. Bioavailability of a single dose of 14 mg ivermectin was determined with AUC_0–t_^T^ of 1701.1 ng/mL h (AUC_0–∞_ of 2382.7 ng/mL h, calculated), C_max_ of 96.2 ng/mL, T_max_ of 4.4 h, and T_1/2_ of 59.9 h. Following 42 mg/day multiple dose (3 × 14 mg every 6 h) administered nasally over 5 days, AUC_0‐∞_ of 2194.4 ng/mL h was analyzed, and 96% of ivermectin concentrations were still measurable 12 h after the last dose. F004 was safe in this study and well‐tolerated. Nine (F004 group) and three (placebo group) of 28 subjects reported 14 symptoms, including a few systemic but mainly local nasal adverse events (AE). The number of subjects reporting AE decreased continuously after both F004 and placebo treatment. All subjects recovered fully with no AE recorded at the end of the study. Nasal examination showed stable patterns of nasal mucosal grading, mucosal bleeding, and crusting of the mucosa. Nasally administered ivermectin is well tolerated in high concentrations and could provide systemic therapeutic benefits in addition to local effects.

## Introduction

COVID‐19 is an infectious disease caused by severe acute respiratory syndrome coronavirus 2 (SARS‐CoV‐2). The WHO reports more than 777 million total cumulative registered coronavirus disease 2019 (COVID‐19) infections and more than 7 million deaths worldwide at the beginning of 2025.^1^ The development of messenger ribonucleic acid (mRNA) vaccines has significantly reduced the number of new infections. Nevertheless, COVID‐19 cases reported to the WHO COVID‐19 dashboard still indicate a great need for effective medication. Main drugs used to treat COVID‐19 are antivirals, immunomodulators, neutralizing antibodies, antithrombotics, and convalescent plasma, which are administered orally, intramuscularly, or subcutaneously.^2^ COVID‐19 transmission occurs through droplet infection, whereby the virus mainly enters the human body via the nasal mucosa. For SARS‐CoV‐2, the membrane‐associated angiotensin‐converting enzyme 2 (ACE2) receptor is the docking site and entry site into nasal mucosa cells. It is therefore conceivable that a nasally applied drug could inhibit the entry of SARS‐CoV‐2 into the nasal epithelium and, if partially absorbed, also have a systemic effect against the virus. Furthermore, in situations where the risk of infection is high, an active ingredient applied nasally shortly beforehand could prevent SARS‐CoV‐2 infection.

Ivermectin was shown in molecular docking studies to shield the spike protein and thereby prevent binding to ACE2.^3^ In addition, ivermectin inhibits SARS‐CoV‐2 replication^4^ and expression of ACE2 and SARS‐CoV‐2 spike protein in cell‐based assays.^5^ Ivermectin binds to the SARS‐CoV‐2 RNA‐dependent RNA polymerase (RdRp), thereby inhibiting viral replication.^4^, ^6^, ^7^ Ivermectin inhibits the nuclear import of virus and host cell proteins through interaction with importin (IMP) heterodimer IMPα/β, which represents a further antiviral effect.^4^, ^5^, ^8^, ^9^, ^10^ Investigator‐initiated trials were performed with ivermectin administered orally to patients with COVID‐19 or for prophylactic use.^11^ Evaluation of the data led to a controversial assessment of the clinical evidence,^12^ possibly due to the route and timing of oral ivermectin administration, the dose, and especially the duration of ivermectin treatment.

A dose of about 54‐times the human dose of Ivermectin suspension 5% nasal spray (company code F004) applied to rats in two non‐clinical studies did not determine any treatment‐related side effect of F004 or vehicle in the nostril, nasal cavity, or pharynx. Quantifiable ivermectin H_2_B_1a_ concentrations could be determined systemically at all blood sampling time points but not in any cerebrum and olfactory bulb samples after sacrifice (studies were performed by HWI pharma services GmbH, data not published).

Nasal application of F004 is aimed to provide dual action against COVID‐19: A local nasal effect to reduce or prevent the viral load at the first hotspot immediately after the outbreak of the infection and a systemic effect, which inhibits the further spread of the virus toward the lungs and other organs. The nasal spray formulation has been developed to apply a significantly high dose of 42 mg/day of ivermectin, which is expected to have a strong local effect. In a first step, we conducted a clinical phase 1 study to investigate the tolerability, safety, and pharmacokinetics of F004 after nasal application in healthy adult subjects.

## Methods

### Clinical Study Design and Ethics

The phase 1 clinical study was prospective, randomized (1:1 F004:placebo), double‐blind, placebo‐controlled, and conducted in two parallel groups of healthy male and female subjects to investigate the nasal tolerability, safety, and systemic exposure of F004 nasal spray containing Ivermectin microsuspension 5%. The trial was carried out between January and March 2023 at the NovaClin Medical Research Center S.R.L. in Timisoara, Romania, and is registered at the European Union Drug Regulating Authorities Clinical Trials Database (EudraCT, Number: 2022‐002670‐82). The study was performed in compliance with Good Clinical Practice (GCP), the Declaration of Helsinki (with amendments), and local legal and regulatory requirements, following approval of the study protocol by the National Agency for Medicines and Medical Devices of the Ministry of Health of Romania. The Clinical Study Protocol, the Subject Information Sheet, and the Informed Consent Form were approved by the appointed Ethics Committee in Bucharest.

The primary outcome is the evaluation of nasal tolerability of F004 subjectively and by nasal inspection with a nasal speculum.

Furthermore, the following secondary objectives were investigated:

1. Systemic exposure of F004 after a single dose application per nose at Baseline (Day 1) and after the last application of multiple dose application on Day 9.
2. Adverse events.
3. Concomitant therapy(ies).
4. General physical examination: body weight, electrocardiogram, blood pressure, heart rate, and body temperature (Day 10/end of study compared to Day 1).
5. Clinical laboratory tests, for example, hematology, clinical chemistry, coagulation (Day 10 compared to Day 1).

### Participants and Randomization

A total of 34 Caucasian subjects (male and female) were screened for the study, of whom 28 subjects were randomized according to the randomization code and allocated to F004 (14 subjects) or a matching placebo of F004 (14 subjects). Six subjects were not included into the clinical trial. A calculation of the sample size power was not performed. Key inclusion criteria were female and male subjects aged ≥18 to ≤60 years at screening visit, able to read and understand the informed consent form, having given written informed consent prior to the performance of any study‐specific procedures, willing and able to apply a nasal suspension, to undergo the investigations, and to follow the visit schedule. Based on the recommendations of the Clinical Trials Facilitation and Coordination Group, women of childbearing potential (WOBCP) must have a negative serum pregnancy test. Women of childbearing potential and males who are sexually active with WOCBP must agree to follow instructions for method(s) of contraception for the duration of treatment with study treatment(s) and for 1 month or 3 months, respectively, after the last dose of study treatment. In addition, male participants must be willing to refrain from sperm donation during this time.

Key exclusion criteria were acute illness, infection or severe chronic disease, positive PCR or Rapid Antigen Test indicative for COVID‐19, nasal polyposis, deviation, perforation, hematoma, superficial vessels, areas of cautery at nasal cavity level, perennial or seasonal rhinitis, known or suspected allergy (hypersensitivity) to the active substance or to any of the excipients, not able to apply the nasal suspension into each nostril thrice daily. Further exclusion criteria were pregnancy or breast‐feeding, clinical laboratory parameter outside the normal range and of clinical significance according to the Investigator, use of any other product to be administered into the nostril, participation in a clinical trial with investigational medicinal products within the last 30 days prior to the start of this trial or any other restrictions due to the participation in other tests/test institutes. Subjects were also excluded with clinically significant abnormal electrocardiogram findings, vital signs, surgery within 4 weeks prior to dosing, history of, or current compulsive alcohol abuse (more than a total of 10 drinks weekly whereby one drink corresponds to 500 mL beer, 200 mL wine, or 50 g spirits); or regular exposure to other substances of abuse, positive urine drug screen, donation or loss of blood equal to or exceeding 500 mL during 90 days before the first administration of study medication, any use of drug, prescribed or OTC (inclusive herbal remedies), within 2 weeks (or within six elimination half‐lives of this medication, whichever is longer) prior to the first administration of study medication except if this will not affect the outcome of the study in the opinion of the clinical investigator.

First, subjects voluntarily signed their informed consent. Screening examinations included physical examination, 12‐lead electrocardiogram, vital signs such as blood pressure, heart rate after 5 min rest, body temperature, and clinical laboratory tests.

### Interventions

Screening medical examinations were performed on Days −14 to −1 before the first F004 nasal spray application on Day 1 (Baseline). From Days 1 to 10, subjects reported to the Medical Research Center on each day for the scheduled dosing or blood sampling procedures. The schedule of the clinical study is given in Table 1.

**Table 1:** Schedule of the clinical study performed with F004.

Parameter	Visits					
Study days	Day −14 to −1 (Screening)	Day 1 (Baseline)	Days 2, 3, 4	Days 5, 6, 7, 8	Day 9	Day 10^j^, ^k^ (EoS)
Written informed consent	x					
Covid‐19 test^a^	x	x	x	x	x	x
Medical history	x					
Physical examination	X^n^					x
Check of inclusion criteria	x	x	x	x	x	x
Check of exclusion criteria	x	x	x	x	x	x
Randomization	x					
Clinical hematology, clinical chemistry, urinalysis	x	x				x
HIV, HBsAg, HCV	x					
Urine drug screen	x	x				
Pregnancy test^c^	x	x				x
Vital signs^m^	x	x				x
12‐lead ECG	x					x
IMP administration^d^		x		x	x	
Blood sampling		X^e^	x^f^	x^g^	x^g^	x^g^
Hospitalization					X^l^	
Local nasal tolerability^h^		x	x	x	x	x
Adverse events^i^	x	x	x	x	x	x
Concomitant medication	x	x	x	x	x	x

^a^ SARS‐CoV‐2 PCR test at screening, Days 1 and 10. A SARS‐CoV‐2 rapid antigen test at screening to Day 10. To avoid any interference with application of the investigational medicinal products (IMP), SARS‐CoV‐2 was determined from throat swab samples on all days of IMP application (Day 1 and Days 5–9) and from nose or throat at screening and from studies on Days 2–4.

^b^ Randomization was done after final screening examination and enrolment decision by investigators.

^c^ For females (in case of positive pregnancy test to be excluded; lactating women were also excluded).

^d^ IMP administration: Day 1 at around 08:00 a.m. Days 5–9, three times daily at around 8:00 a.m., 2:00 p.m., and 8:00 p.m.

^e^ Blood sampling (single dose): Pre‐dose (within 30 min) and post‐dose at 0.33, 0.67, 1.00, 1.50, 2.00, 3.00, 4.00, 5.00, 6.00, 8.00, and 12.00 h.

^f^ Blood sampling: Post‐dose at 24 h (Day 2), 48 h (Day 3), and 72 h (Day 4).

^g^ Blood sampling: Post‐dose at 96 h (Day 5); (identical to pre‐dose blood sample of the morning dose on Day 5) Blood sampling (multiple dose) Day 5: Pre‐dose at around 8:00 p.m.; Days 6–8: Pre‐dose at around 8:00 a.m. and 8:00 p.m. Day 9: Pre‐dose at around 8:00 a.m. and 8:00  p.m. (last dose of IMP) and thereafter post‐dose at 0.33, 0.67, 1.00, 1.50, 2.00, 3.00, 4.00, 5.00, 6.00, 8.00 and 12.00 h.

^h^ Local nasal tolerability was assessed by investigators and subjects before and 15 min and 1 h after each dose on Day 1 and Days 5–9 (on Day 1, the evaluation was not done before administration). On Days 2–4 and Day 10, assessment was done at the scheduled times for blood sampling. Further assessment was possible at any time during the entire study period. Local inspection by nasal speculum by investigators: before and 1 h after administration on Day 1 and before and 1 h after administration of the morning and evening dose on Days 5–9, and Day 10 (final safety examination).

^i^ Questioning for adverse events: during screening, on Day 1 before and 1.0, 4.0, 8.0 and 12.0 h after drug administration, on Days 2–4 before blood sampling in the morning, on Days 5–8 before and 1.0, 4.0, 8.0 and 12.0 hours after drug administration in the morning and on Day 9 before and 1.0, 4.0, 8.0, 12.0, 13.0, 16.0 and 24.0 h after drug administration in the morning. On Day 10, during final examinations.

^j^ End of the study at Day 10 or premature termination at any time. End of study (EoS) examinations.

^k^ If any serious adverse event occurred, the subject was to be followed until recovery without sequelae.

^l^ Subject was confined to the clinical unit on Day 9 from the morning at around 8:00 a.m. until after the last blood sampling on Day 10 and after the final end of study examination.

^m^ Vital signs included heart rate and blood pressure (in sitting position; after 5 min rest) and body temperature.

^n^ Physical examination included ear‐nose‐throat (ENT) specialist examination with a speculum for detection of any nasal polyposis, deviations, perforations, hematoma, and superficial vessels at the nasal cavity level.

To assure the safety of all persons involved in the clinical trial, a SARS‐CoV‐2 PCR test was performed at screening, Days 1 and 10 and a SARS‐CoV‐2 Rapid Antigen Test at screening, and from Days 1 to 10. To avoid any interference with application of study medication, SARS‐CoV‐2 was determined from throat swab samples on all days of application of study medication (Day 1, Days 5–9) and from the nose or throat at screening and from study Days 2 to 4.

On Day 1 and the days of three‐times‐a‐day dosing (Days 5–9), subjects remained in the clinical unit for approximately 13 h until all blood samples were taken, and examinations were completed. On Day 9, the subjects were accommodated in the clinical unit until the next day (Day 10). On Day 10, subjects were discharged after the last blood sample and after having successfully undergone all final examinations as performed at screening.

Standard hospital meals were provided to the subjects in the clinical unit on Day 1 (4 and 10 h after nasal spray application) and on Days 5, 6, 7, 8, and 9.

### Treatments

The Sponsor (HWI pharma services GmbH) of this clinical phase 1 study has developed F004 as a nasal spray, containing Ivermectin suspension 5%. The aim was to develop a high‐concentrated active pharmaceutical ingredient suspension to keep the required application volume low while maintaining a high stability over the planned storage duration. The ivermectin 5% concentration turned out to be ideal, fulfilling both requirements at once.

A single dose (one actuation) of F004 nasal spray delivers 140 µL of nasal formulation. Per actuation, 7 mg ivermectin is applied per nostril, accounting to 14 mg/nose, about 0.23 mg/kg/nose for a human of 60 kg body weight. Thrice daily application to the nose corresponds to a daily dose of 42 mg ivermectin per subject, accounting for about 0.7 mg/kg/day for a human of 60 kg body weight.

After training on how to apply the nasal spray, one actuation was performed per nostril of the test product F004 or the control product placebo according to the randomization code. The investigational medicinal product was applied by the subject himself/herself in a sitting position at the clinical unit under the supervision of an investigator: at 8.00 a.m. on Day 1, and at 8.00 a.m., 2.00 p.m., and 8.00 p.m. on Days 5, 6, 7, 8, and 9. The wash‐out period was 96 h after single‐dose application on Day 1 and before multiple‐dose spray administration on Day 5.

### Assessment of Local and Systemic Tolerability and Safety

#### Local Nasal Tolerability

Local nasal tolerability was assessed by both investigators and subjects at the following time points: before (not on Day 1), as well as 15 min and 1 h after each nasal spray application. Assessments included a total of 51 time points: five (5) following single dose and 46 after multiple dose 3 times/day applications. If the number of time points is multiplied by the number of subjects treated (n = 28), there is an overall number of 1428 time points, that is, 714 each after F004 and placebo.

Global local tolerability was assessed by means of a 5‐point scale categorized as follows: 1 = very good; 2 = good; 3 = fair; 4 = poor; 5 = very poor. Acceptable scores were the scores of 1, 2, or 3.

#### Nasal Inspection

An ear‐nose‐throat (ENT) specialist performed the nasal inspection in the clinical unit using a nasal speculum before and 1 h after each application of the morning and the evening dose, and on Day 10 as part of the end of study (EoS) examinations. The following symptoms were assessed using appropriate scales:

Nasal mucosal grading scale: Grade 0 = no abnormal findings; Grade 1A = focal nasal mucosal irritation (inflammation, erythema, or hyperemia); Grade 1B = superficial nasal mucosal erosion; Grade 2 = moderate nasal mucosal erosion. Mucosal bleeding and crusting of mucosa scale: 0 = none; 1 = mild; 2 = moderate; 3 = severe.

#### Adverse Events and Concomitant Medication

Questioning for adverse events (AE) was done and concomitant medication was checked on Day 1 before and 1.0, 4.0, 8.0, and 12.0 h after nasal spray application, on Days 2, 3, and 4 at the time points when the subjects came to the clinical unit for blood sampling and on Days 5, 6, 7, 8, 9, and Day 10 before and 1.0 h after each dose.

In addition, spontaneous reporting of AE by any subject was possible at any time up to the EoS examination on Day 10. If any serious adverse event (SAE) would have occurred, the subject was to be followed until recovery without sequelae.

All AE were to be recorded, whether considered non‐serious or serious, drug‐related or not.

#### Other Safety Examinations

A general medical examination was performed at screening and Day 10, including a physical examination and 12‐lead ECG. Vital signs were measured at screening, Days 1, 5, and 10, that is, heart rate and blood pressure (in sitting position; after 5 min rest) and body temperature.

Clinical laboratory tests (hematology, clinical chemistry, coagulation, and urinalysis) were performed at screening, Days 1 and 10, and a drug screen at screening and Day 1. Abnormal laboratory findings considered to be clinically significant were to be recorded as adverse events.

#### Pharmacokinetic Evaluation and Times of Blood Sampling

Following mean pharmacokinetic parameters were evaluated for systemic exposure after first application on Day 1: Area under the plasma concentration–time curve (AUC) from the first time point (t = 0) to the time point of the last measured concentration (t_last_) at 96 h after application (AUC_0–t_^T^), AUC (t = 0) to infinity (∞) (AUC_(0–∞)_), maximum plasma concentration (C_max_), time of maximum plasma concentration (T_max_), elimination half‐life (T_1/2_), and elimination rate constant (λ_z_).

For the evaluation of the pharmacokinetics after the last systemic application on Day 9, the following parameters were additionally analyzed: AUC during a dosage interval at steady state (after evening dose [= last dose] on Day 9) (AUC_(0–τ)_), C_max_ at steady state (C_max,ss_), concentration at the end of a dosing interval at steady state (C_τ,ss_), minimum plasma concentration at steady state (C_min,ss_), T_max_ at steady state (T_max,ss_), peak‐trough fluctuation (PFT), and peak‐trough ratio (PTR).

AUC_(0–t)_, AUC_(0–∞)_, and AUC_(0–τ)_) were calculated using the trapezoidal rule. T_1/2_ was calculated from concentrations of the elimination phase using semi‐log transformed data and linear regression. λ_z_ was calculated by log‐linear regression of concentrations observed during the terminal phase of elimination. All other pharmacokinetic parameters were directly obtained from measured values. PTF was calculated with (C_max,ss_ – C_min,ss_)/C_av,ss_ with C_av,ss_ = AUC_(0‐τ)_/τ. PTR was obtained by C_max,ss_ /C_min,ss_.

Ivermectin B1a (ivermectin) concentrations were analyzed in plasma using a validated liquid chromatography‐mass spectrometry/mass spectrometry (LC‐MS/MS) method after solid phase extraction. Good Laboratory Practice (GLP)^13^ and GCP guidelines^14^ were considered, and the reflection paper for laboratories that perform the analysis or evaluation of clinical trial samples.^15^

Quantification of ivermectin in plasma was carried out using a ReproSil‐Pur C18‐AQ HPLC column (3 µm, 75 × 2 mm) with a SecurityGuard C18 pre‐column (4 × 2 mm). Mobile phases were: Solvent A: water/0.5 M ammonium formiate/formic acid (1000:25:1, v/v/v), Solvent B: methanol/acetonitrile (1:1, v/v). Flow rate was 0.35 mL/min (linear gradient). MS/MS‐detection: electrospray ionization was carried out at an ionspray voltage of 5500 V and a temperature of 350°C.

Lower limit of quantification (LLOQ) was 0.25 ng/mL. Calibration range included 0.25–250.00 ng/mL.

Blood samples were collected for analysis of ivermectin B1a (hereinafter referred to as ivermectin) plasma concentrations on Day 1 (single dose nasal application) before (within 30 min) and after nasal spray application at 0.33, 0.67, 1, 1.5, 2, 3, 4, 5, 6, 8, and 12 h, on Day 2 at 24 h, on Day 3 at 48 h, on Day 4 at 72 h, on Day 5 at 96 h (identical to pre‐dose blood sample of the multiple dosing morning dose on Day 5), on Days 5 to 8 (multiple dosing) pre‐dose at around 8:00 and 20:00, on Day 9 pre‐dose at around 8:00 and 20:00 h (last dose) and thereafter post‐dose at 0.33, 0.67, 1, 1.5, 2, 3, 4, 5, 6, 8 and 12 h.

### Statistical Safety Evaluation

All data of any safety parameter investigated were tabulated, where applicable, descriptive group statistics (mean, standard deviation, minimum, maximum, and number of valid cases) were performed.

Clinical laboratory tests outside the normal range were clinically evaluated.

## Results

A total of 28 subjects (15 male and 13 female) participated in the study, aged 18 to 58 years and body weight between 43 and 104 kg. Fourteen healthy female and male subjects each were treated with F004 or placebo of F004 nasal spray of whom 27 subjects completed the study. One subject of the placebo group was withdrawn due to a common cold before the start of multiple dosing on Day 5.

**Figure 1:**
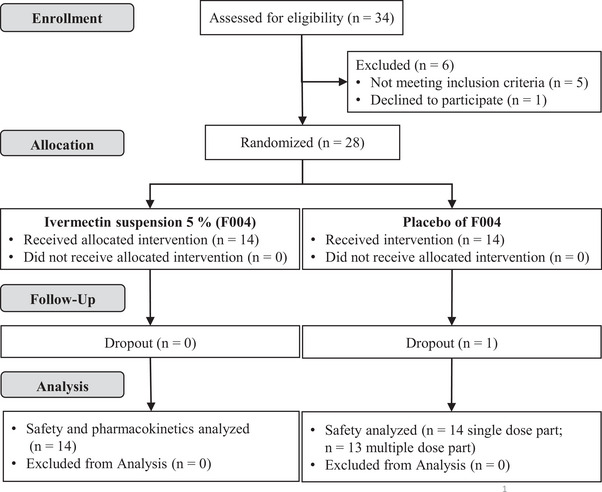
Disposition of subjects

All 14 subjects treated with F004 were included in the pharmacokinetic analysis of ivermectin (IVM) plasma levels, and all 28 subjects into the safety analysis. One subject treated with a placebo dropped out due to a common cold on Day 5 and missed the multiple‐dose treatment. Therefore, a total of 27 subjects (n = 14 F004, n = 13 placebo) completed the entire clinical study (Figure 1).

The age of the 28 Caucasian subjects (15 male and 13 female) was between 18 to 58 years (mean: 33.5 ± 10.7 years), body weight was between 43 to 104 kg (mean: 75.4 ± 13.5 kg) and the mean body mass index was between 19 to 30 kg/m^2^ (mean: 25.7 ± 3.4kg/m^2^). Homogeneity analysis of age and sex did not show any significant differences between the two treatment groups (Table S1).

### Nasal Tolerability

**Figure 2:**
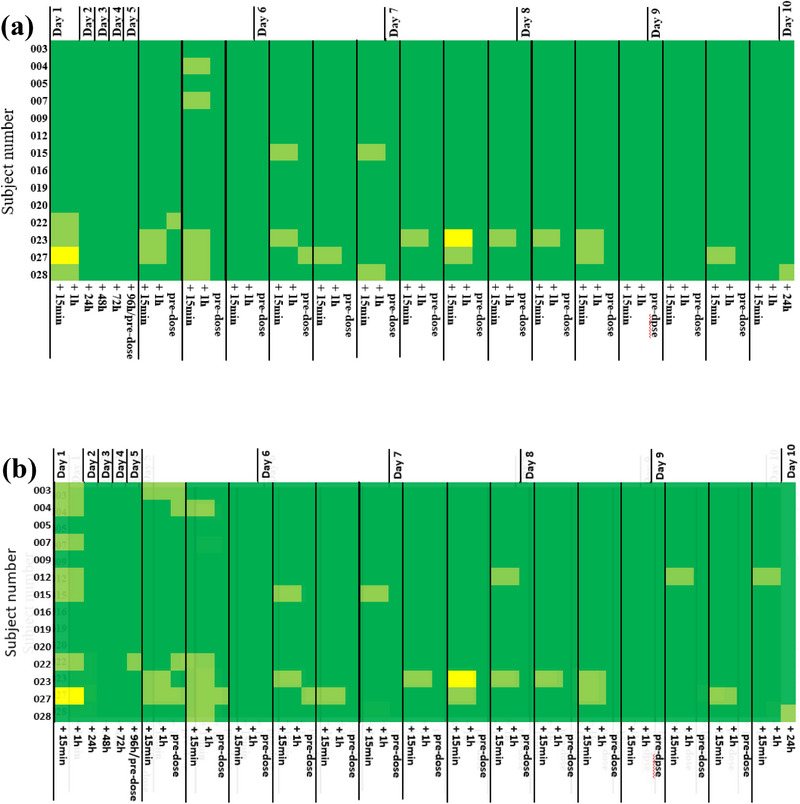
Nasal tolerability of F004 at all time points assessed by the investigator (a) and by subjects (b). Score: 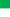 very good, 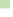 good, 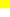 fair, 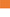 poor, 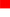 very poor.

F004 application was assessed as very good by the investigator in 50% of the 14 subjects at all 714 time points. Nasal tolerability was rated as good (47/714) or fair (4/714) at 51 of 714 time points in seven out of the 14 subjects. Of these subjects, the maximum duration of assessment good was 5 days of the 10 investigational days reported for two subjects. Of 51 possible assessment time points per subject, the highest number was 16, which was rated as good for one subject. The rating, poor or very poor, was not reported. The number of good and fair assessments decreased in favor of very good during the 10‐day period, with only one subject each, assessed as good on Days 9 and 10 (Figure 2a).

Overall assessment results were slightly different when rated by the subjects themselves, expressed by the rating very good by 36% (n = 5) of the 14 subjects at all 714 time points. The rating poor or very poor was not reported (Figure 2b).

**Figure 3:**
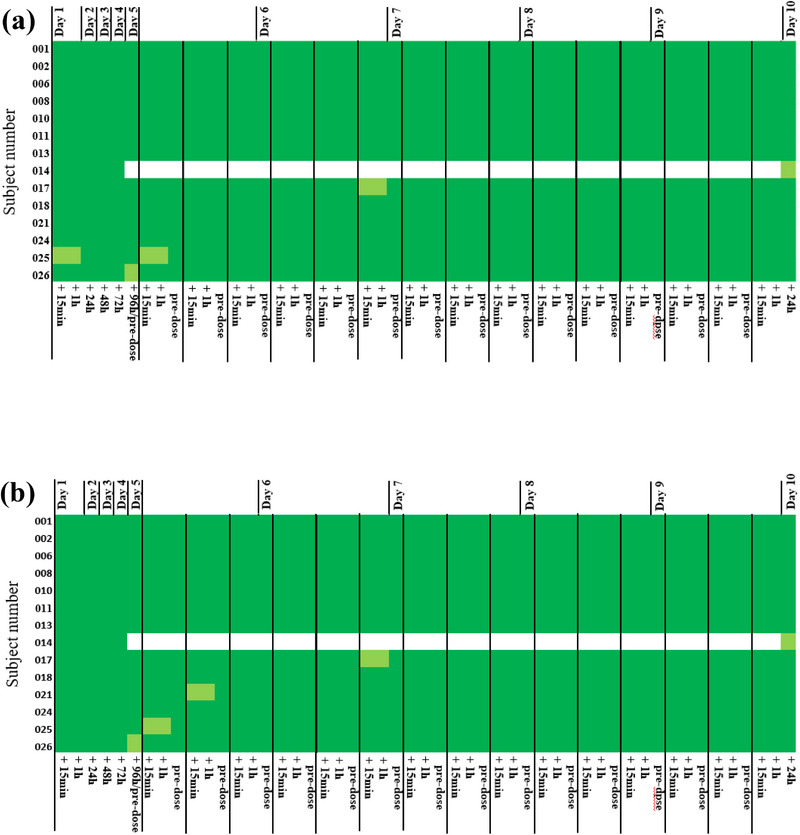
Nasal tolerability of the placebo of F004 at all time points assessed by the investigator (a) and by subjects (b). Subject No. 014 was withdrawn due to a common cold on Day 5 of the study. No assessment was performed from Days 5–9 shown in white. Assessment was carried out on Day 10 during the final examination. Score: 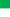 very good, 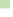 good, 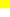 fair, 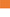 poor, 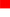 very poor.

Placebo application was assessed as very good by the investigator in 79% of the 14 subjects at all 714 time points and as good at 7 of 714 time points in 3 of the 14 subjects. The maximum duration of good ratings was 2 days for one subject and 1 day each for two subjects out of a total of 10 investigational days. Of 51 possible assessment time points per subject, the highest number was 4, which was rated as good for one subject. From Day 7 onward, nasal tolerability was rated as very good at all time points for all subjects (Figure 3a).

Overall assessment results were similar when rated by the 14 subjects themselves, with the rating very good by 71% (n = 10) of the subjects at all 714 time points and rating very good from Day 7 onward by all subjects (Figure 3b).

### Nasal Inspection

#### Nasal Mucosal Grading

**Figure 4:**
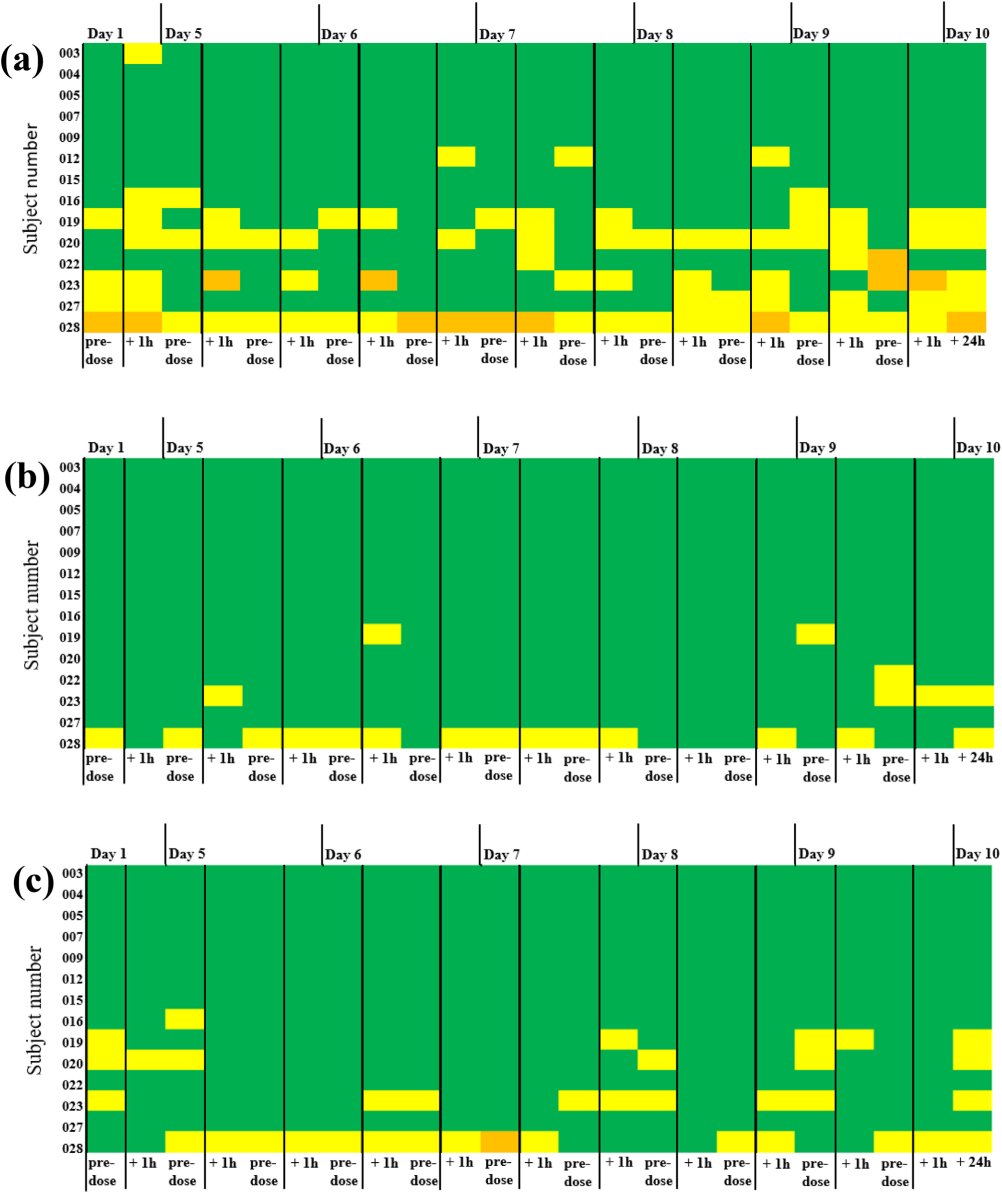
Nasal inspection after F004 application. (a) Nasal mucosal grading: 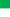 Grade 0 (no abnormal findings), 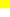 Grade 1A (focal nasal mucosal irritation), 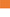 Grade 1B (superficial basal mucosal erosion), 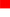 Grade 2 (moderate nasal mucosal erosion). (b) Nasal mucosal bleeding and (c) nasal mucosal crusting: 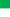 Grade 0 (none), 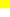 Grade 1 (mild), 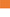 Grade 2 (moderate), 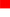 Grade 3 (severe).

**Figure 5:**
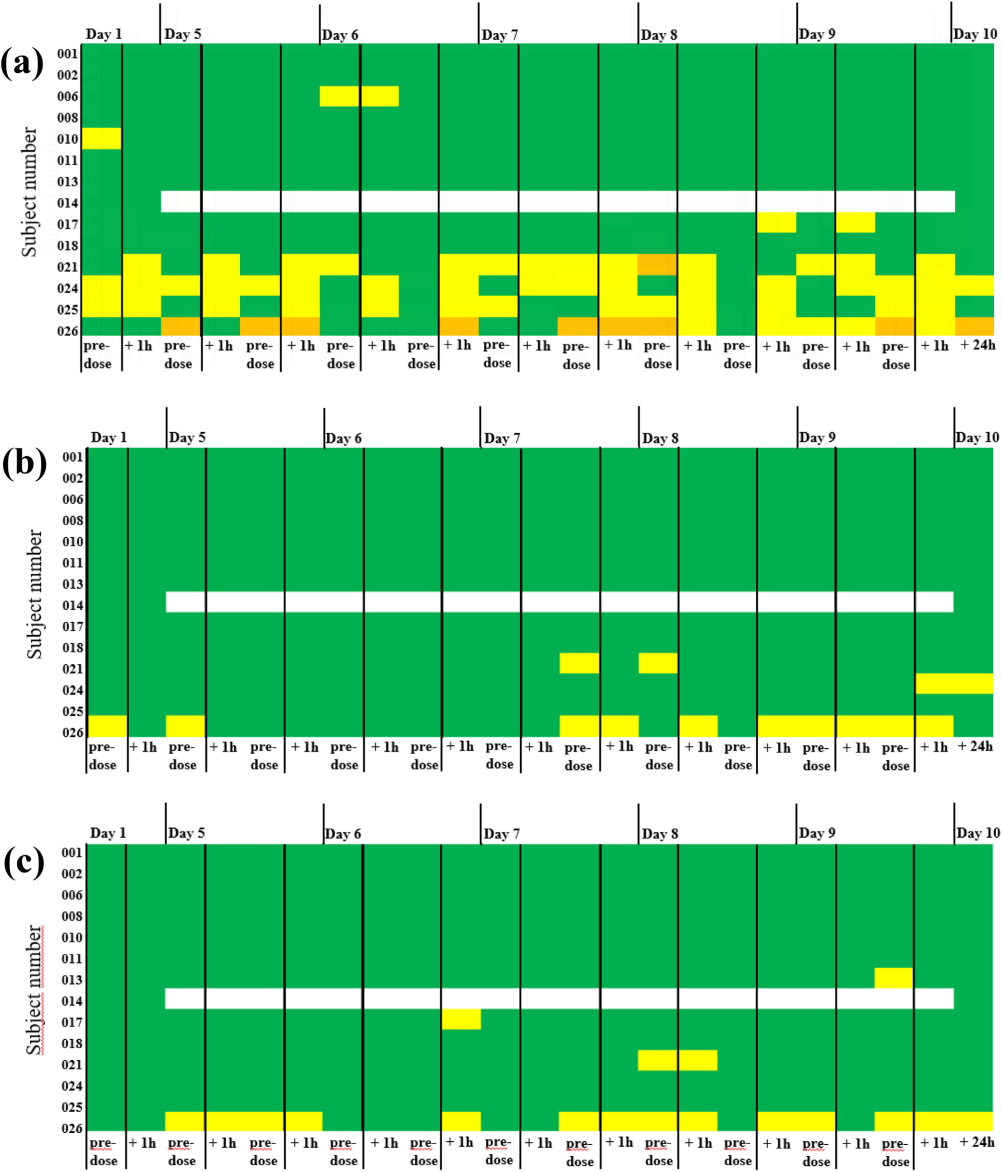
Nasal inspection after placebo of F004 application. Subject no. 014 was withdrawn due to a common cold on Day 5 and was not assessed from Days 5–9 shown in white, but on Day 10 (final examination). (a) Nasal mucosal grading: 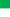 Grade 0 (no abnormal findings), 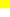 Grade 1A (focal nasal mucosal irritation), 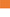 Grade 1B (superficial basal mucosal erosion), 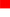 Grade 2 (moderate nasal mucosal erosion). (b) Nasal mucosal bleeding and (c) nasal mucosal crusting: 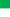 Grade 0 (none), 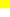 Grade 1 (mild), 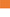 Grade 2 (moderate), 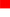 Grade 3 (severe).

Pattern of nasal mucosal grading was stable and did not deteriorate in any subject during the 10‐day investigational period following F004 and placebo application, as shown in Figures 4a and 5a.

After F004 and placebo application, five (5) and seven (7) subjects were continuously evaluated with Grade 0 throughout Days 1 to 10. Four (4) subjects and three (3) subjects were already diagnosed with Grade 1A (n = 6) or Grade 1B (n = 1 F004 group) before the first F004 or placebo nasal spray application.

No subject was assessed Grade 2 at any time point. The other 16 subjects occasionally switched back and forth between Grade 0 and grade 1B. After F004 or placebo application, eight (8) and seven (7) subjects were examined at some time points with Grade 1A, and three (3) and two (2) subjects with Grade 1B (Figures 4a and 5a).

#### Nasal Mucosal Bleeding

The pattern of nasal mucosa bleeding was stable and did not deteriorate in any subject between Days 1 and 10 after the application of F004 and placebo.

All subjects were assessed as Grade 0 (none) and Grade 1 (mild), and none with Grade 2 (moderate) or Grade 3 (severe). 10/14 subjects each of F004 and placebo (including Subject No. 14 until termination of participation) were continuously assessed Grade 0 throughout Days 1 to 10, whereas the other subjects occasionally switched back and forth between Grade 0 and Grade 1. Mild bleeding in one subject each following F004 and placebo treatment at pre‐dose at Day 1 did not deteriorate and was not determined at all time points (Figures 4b and 5b).

#### Nasal Mucosal Crusting

The pattern of nasal mucosal crusting was stable and did not deteriorate in any subject between Days 1 and 10 after application of F004 and placebo.

All subjects were assessed as Grade 0 (none) and Grade 1 (mild), and no subject with Grade 2 (moderate) or Grade 3 (severe). 9/14 subjects treated with F004 and 10/14 subjects treated with placebo (including Subject No. 14 until termination of participation) were continuously assessed Grade 0 throughout Days 1 to 10. The other subjects occasionally switched back and forth between Grade 0 and Grade 1, with one exception, as one subject with mild bleeding already at pre‐dose on Day 1 was assessed Grade 3 at a single time point on Day 7 before the F004 morning dose. Furthermore, three (3) of the 14 subjects treated with F004 were already diagnosed with mucosal crusting before the first nasal spray application on Day 1, but none of the subjects treated with placebo (Figures 4c and 5c).

#### Adverse Events

**Figure 6:**
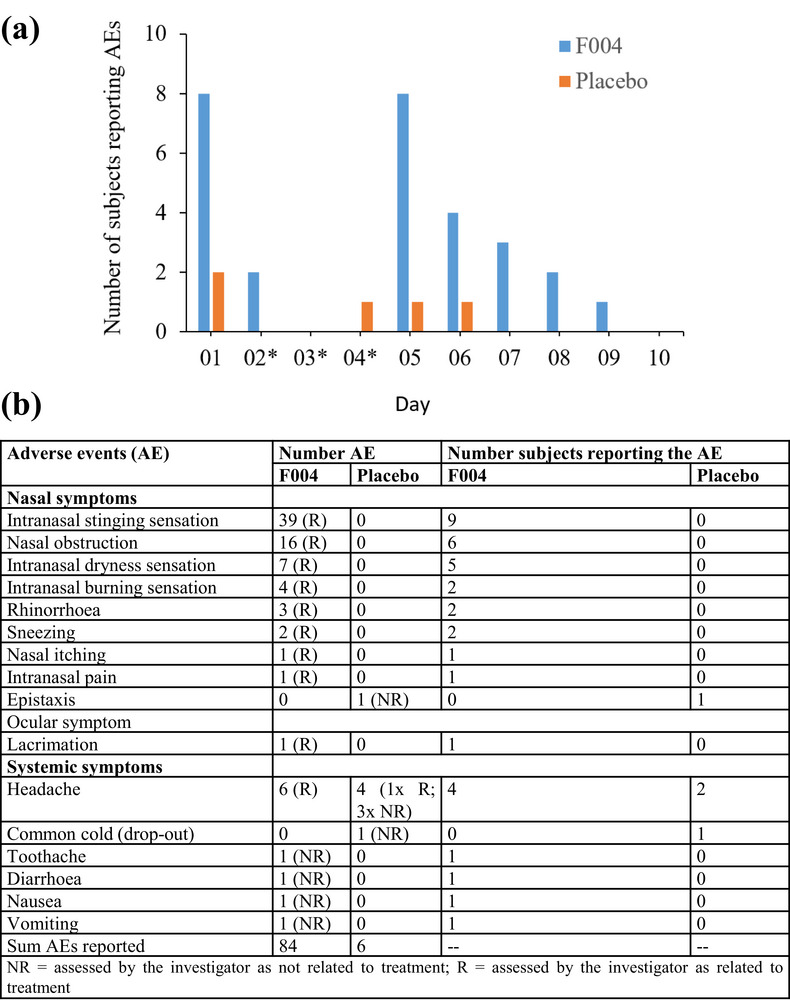
(a) Number of reported adverse events (AE) per treatment visit. *Days without F004 or placebo application, but with blood sampling and AE recording. (b) Evaluation of reported adverse events (AE).

Treatment with F004 led to a total of 84 adverse events (AE) in 9 of 14 subjects, which could be attributed to 14 symptoms. Of these, eight symptoms were local nasal AE, and five symptoms were systemic AE. In addition, one subject reported an ocular AE (Figure 6b). Nasal symptoms were dominated by an intranasal stinging sensation in nine subjects and nasal obstruction in six subjects. Systemic symptoms were dominated by headache in four subjects. Three symptoms reported after placebo of F004 application were one local nasal AE (epistaxis) and two systemic AE (headache and common cold (one subject, drop‐out) (Figure 6b). All observed AE were non‐serious and of mild (58 AE) or moderate intensity (32 AE). All subjects recovered fully with no AE recorded in any subject at the EoS examination on Day 10 (Figure 6a). Local nasal and ocular symptoms were assessed as related (possible or probable) to F004, with the exception of intranasal pain, assessed as improbable related; the local nasal symptom epistaxis was assessed as improbable related to placebo. Systemic symptoms and other non‐nasal local symptoms reported after F004 or placebo application were assessed as improbable (headache, diarrhea, nausea, toothache), unknown (vomiting), or not related (common cold). Headache was assessed as possible related to the placebo in one subject. The number of subjects reporting AE was highest on Day 1 (nasal spray single dose) and Day 5 (first day of nasal spray multiple doses), with eight subjects each reporting AE after F004 and two (2) and one (1) subject, respectively, after placebo application. Thereafter, the number of subjects reporting AE decreased continuously after both F004 and placebo (no reports from Day 7 onward) treatment, and thus also the number of AE recorded. No AE were reported on Day 10, 12 h after the last nasal spray administration and subsequent EoS examination. The highest number of adverse events reported by a single subject was 22, with seven different AE/symptoms.

#### Pharmacokinetics

**Figure 7:**
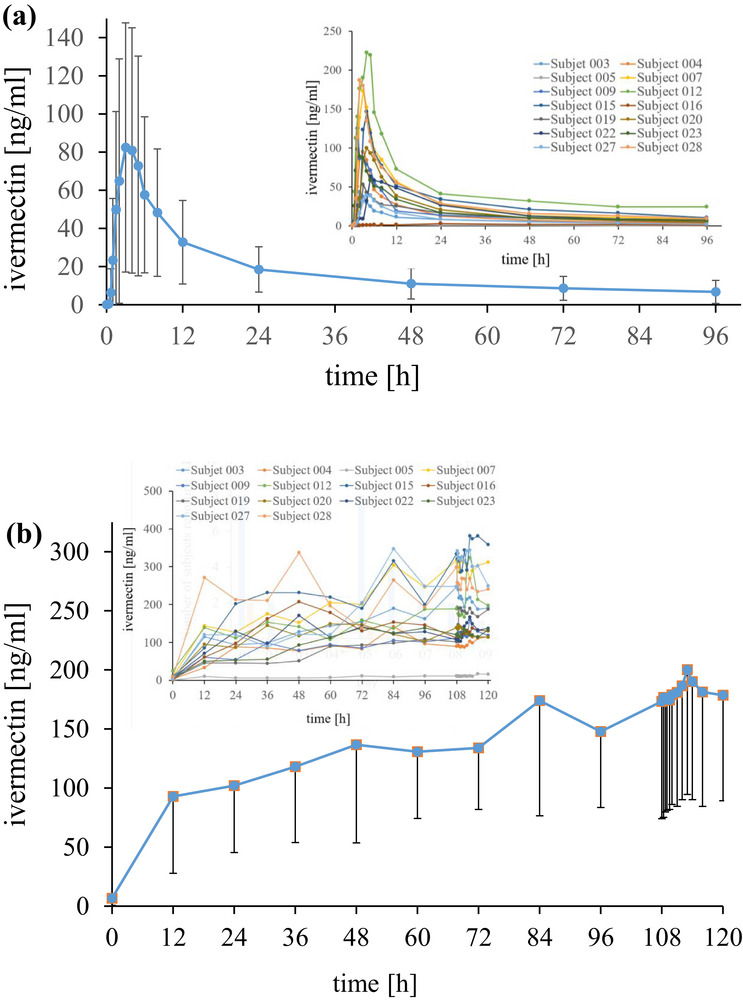
Pharmacokinetics after (a) single dose application and (b) multiple dose application of F004. Ivermectin concentrations before each morning and evening application from Days 5–9. Inserts in (a) and (b) show the respective individual courses of ivermectin concentrations.

Pharmacokinetics of F004 were determined over 96 h (AUC_0‐t_^T^) following a 14 mg/day single dose and over 12 h after 42 mg/day multiple nasal dose (3 × 14 mg every 6 h for 5 days; AUC_0–τ_). Results are given in Table 2. Figure 7a shows the mean and individual course of ivermectin plasma concentrations after 14 mg single dose application. Mean value for AUC_0–t_^T^ was determined as 1 701.1 ng/mL h. Mean value of AUC_0–∞_ was calculated as 2382.7 ng/mL h, mean C_max_ was 96.2 ± 68.1 ng/mL at 4.4 h (T_max_) with standard deviation (SD) of ± 3.4 h and a coefficient of variation (CV%) of 78%. Thereafter, mean ivermectin concentrations continuously decreased to 80.9 ± 64.3, 57.6 ± 40.9, 48.3 ± 33.4, and 32.8 ± 1.9 ng/mL at 4, 6, 8, and 12 h after nasal application. At 6, 8, and 12 h after F004 single dose, ivermectin concentrations decreased to 72%, 59%, and 41% compared to ivermectin concentrations at T_max_ at 4 h (100%). Individual C_max_ concentrations ranged from 1.5 ng/mL at 12 h (latest T_max_) after nasal application to 221.9 ng/mL at 4 h after application. The earliest T_max_ was reached after 1.5 h with C_max_ of 125.0 ng/mL. At 96 h after nasal application, concentrations were determined with a mean of 6.7 ng/mL (median: 6.1 ng/mL), a minimum of 0.3 ng/mL and a maximum of 24.7 ng/mL, that is, at pre‐dose of 5‐day multiple dosing. This course of ivermectin concentrations corresponds to a mean T_1/2_ of 59.9 h and median T_1/2_ of 57.7 h. Figure 7b shows the mean and individual course of the ivermectin concentrations following 5‐day multiple dosing. Mean steady state ivermectin concentrations seem to be reached at 72 h (Day 8). Mean C_min,ss_ (CV%) seems to stabilize from 48 h (Day 7, morning) with mean values of 136.7 ng/mL ± 83.1 ng/mL (61%) and 147.8 ng/mL ± 64.3 ng/mL (44%) at 96 h (Day 9, morning). Mean values for AUC determined for 12 h after the last (evening) dose on Day 9 (AUC_0‐τ_) were analyzed as 2 194.4 ng/mL h ± 1 125.8 ng/mL h, and C_max,ss_ as 213.3 ng/mL ± 106.9 ng/mL. Accumulation of ivermectin is determined during this 5‐day study period, with a factor calculated to be 1.3. Individual ivermectin concentrations show low plasma levels determined after a single dose and 5‐day multiple dose application in one subject. Another subject revealed low plasma levels only after single dose but not after multiple dose application. At 2, 4, 6, 8, and 12 h after the last multiple dose F004 nasal application on Day 9, mean values of ivermectin ± SD and (CV%) were determined as 179.0 ± 92.9 ng/mL (52%), 186.5 ± 96.4 ng/mL (52%), 190.0 ± 99.9 ng/mL (53%), 181.2 ± 96.8 ng/mL (53%), and 178.6 ± 89.4 ng/mL (50%) (Figure S1). This accounts to a factor of 2.8, 2.3, 3.3, 3.6, and 5.5 of mean ivermectin concentrations after F004 multiple dose application compared to the single dose nasal administration on Day 1. Mean pre‐dose value was 173.3 ± 99.3 ng/mL (57%), that is, ivermectin concentrations 6 h after the 2:00 p.m. dose. Compared to concentrations at T_max,ss_ (= 100%) at 4.8 ± 3.9 h (81%), ivermectin concentrations were stable and still 96% of T_max,ss_ at 12 h after the last F004 dose.

**Table 2:** Pharmacokinetic Parameters of Ivermectin

Pharmacokinetic parameters	Ivermectin dose (fasted)	Ivermectin dose
Mean values ± SD ± CV (%)	F004 14 mg single dose	F004 14 mg^a^ multiple dose
AUC_0–t_^T^ (ng/mL h) (CV%)	1 701.1 ± 1 178.2 (69)	na
AUC_0–∞_ (ng/mL h; calculated) (CV%)	2 382.7 ± 1 929.5 (81)	na
AUC_0–τ_ (ng/mL h) (CV%)	na	2 194.4 ± 1 125.8 (51)
C_max_ (ng/mL) (CV%)	96.2 ± 68.1 (71)	na
C_max,ss_ (ng/mL) (CV%)	na	213.3 ± 106.9 (50)
C_min,ss_ (ng/mL) (CV%)	na	153.1 ± 80.5 (53)
C_t,ss_ (ng/mL) (CV%)	na	178.6 ± 89.4 (50)
T_max_ (h) (CV%)	4.4 ± 3.4 (78)	na
T_max,ss_ (h) (CV%)	na	4.8 ± 3.9 (81)
T_1/2_ (h) (CV%)	59.9 ± 20.1 (34)	na
λ_z_ (h^−1^)	0.013 ± 0.004	na
PTF (%)	na	34.2 ± 15.0 (44)
PTR	na	1.4 ± 0.2 (17)
Concentrations: 4 h pa (ng/mL)	80.9 ± 64.3 (79)	186.5 ± 96.4 (52) [2.3^b^]
Concentrations: 6 h pa (ng/mL)	57.6 ± 40.9 (71)	190.0 ± 99.9 (53) [3.3^b^]
Concentrations: 8 h pa (ng/mL)	48.3 ± 33.4 (69)	181.2 ± 96.8 (53) [3.6^b^]
Concentrations: 12 h pa (ng/mL)	32.8 ± 1.9 (67)	178.6 ± 89.4 (50) [5.5^b^]

na = not applicable; pa = post application (after application).

*Note*: Analyte: ivermectin B1a, active pharmaceutical ingredient of F004 5%.

AUC_0–t_^T^ = AUC determined for 96 h.

AUC_0–τ_ = AUC determined for 12 h after the last (evening) dose on Day 9 after multiple dose application three times per day for 5 days.

Data are shown as arithmetic mean values ± standard deviation (SD) and coefficient of variation (CV%).

^a^ F004 14 mg multiple dose: applied three times/day for 5 days (42 mg/day) on Days 5 to 9; steady state pharmacokinetics after the last/evening dose on Day 9 with 12‐h blood sampling period and every 12 h during the five dosing days (Days 5 to 9).

^b^ Indicates the factor of ivermectin B1a concentrations after 5‐day multiple dosing compared to the single dose.

## Discussion and Conclusions

Ivermectin was first approved in 1987 as a highly effective antihelminthic agent for the treatment of onchocerciasis. Tablets are also used to treat scabies, strongyloidiasis, and lymphatic filariasis, among others, and a 1% cream for rosacea.^16^ Different amounts of ivermectin are administered depending on the indication. For example, 150 µg/kg is administered once a year for the treatment of onchocerciasis.^17^ Larger quantities of 300 to 400 µg/kg once a year are used for the treatment of lymphatic filariasis.^18^ Effect of ivermectin on COVID‐19 patients was investigated in several studies, whereby the dosages and the treatment periods differed significantly in some cases. As the results were partly contradictory, the clinical efficacy of ivermectin in COVID‐19 patients has not been conclusively clarified.^12^ One possible reason for this could be insufficient dosage and bioavailability after oral administration.

The nasal epithelium is the site of SARS‐CoV‐2 infection and subsequent viral replication. It therefore makes sense to apply ivermectin nasally. For this reason, a nasal spray (F004) containing an ivermectin microsuspension 5% has been developed that can be used to administer very high doses locally that cannot be achieved systemically.

Per actuation, F004 nasal spray applies 7 mg ivermectin per nostril, accounting for 14 mg/nose. Three times per day, nasal application corresponds to ivermectin 42 mg/day per subject. Over a period of 10 days, a total of 224 mg ivermectin were applied nasally, that is, a single dose of F004 14 mg (Day 1) and multiple doses administered three times per day (42 mg/day) for 5 days (210 mg from Days 5–9) after a washout period of 96 h (4 days, Days 2–5).

F004 or a matching placebo of F004 was considered safe both locally and systemically and well tolerated by the subjects after both single use and multiple applications over a period of 5 days.

Nasal examination was performed with a nasal speculum by an ear‐nose‐throat specialist. Pattern of nasal mucosal grading, mucosal bleeding, and crusting of mucosa was stable and did not deteriorate in any subject during the 10‐day investigational period following F004 and placebo topical application. This also applies to proven cases with pre‐existing impairment of the nasal mucosa, that is, mild bleeding or crusting. Nasal tolerability assessment results evaluated by the investigator and subjects corresponded to the ones evaluated with a nasal speculum and vice versa.

Nine of fourteen (9/14) subjects treated with F004 and 3/14 subjects treated with placebo reported treatment‐emergent adverse events. With one exception, the local (nasal and ocular) symptoms were assessed as possible or probable related to treatment with F004, whereas the systemic symptoms and one nasal symptom were assessed as improbable related or unknown. After placebo administration, common cold and epistaxis were assessed as not or improbable related to placebo, but headache as possible related in one case.

Nasal symptoms are consistent with those most commonly reported as adverse reactions after topical treatment of patients with rosacea, that is, skin burning sensation, skin irritation, pruritus, and dry skin.^19^ Systemic AE are also in accordance with the ones reported in other clinical studies performed with ivermectin tablets in healthy subjects.^20^

The adverse events reported, the pattern and course of adverse events correspond to the nasal tolerability results evaluated by the Investigator and subjects themselves, and the ones evaluated by the ENT specialist with a nasal speculum.

The number of subjects reporting AE was highest on Day 1 (single dose) and Day 5 (first day of multiple doses). Thereafter, the number of subjects reporting AE and the number of AE recorded decreased continuously after F004 and placebo. No subject reported any AE at Day 10 at the final examination at about 12 h after the last treatment with F004 or placebo.

Increase in the number of AE reported per subject on Day 5 compared to Day 1 after F004 application may be attributed to the increase in the number of F004 applications from one dose per day on Day 1, no nasal spray application on Days 2–4, but three doses per day on Days 5–9, with more frequent questioning for AE.

The increases in local tolerability assessments were very good, and the decrease in subjects reporting AE and the number of AE recorded toward the middle or end of the investigational 10‐day period are remarkable, as they occurred during the 5‐day multiple dose three times daily nasal application of both treatments. One possible explanation is that the subjects were familiar with the (a) nasal applications during the clinical study, especially if only minor inconveniences occurred throughout the investigational period, (b) stressful situation during participation in a phase 1 clinical trial with first application of a newly developed nasal spray, (c) inability to maintain the daily routine and habits, and (d) generally high tolerability of the nasal spray containing F004 or placebo.

A correlation was not determined between the total number of AE recorded by a subject and with pharmacokinetic parameters.

Based on the pharmacokinetic results of this study, the plasma concentrations of ivermectin over time after single and multiple nasal administration of F004 can be described and evaluated, see Table 2, Figure 7, and Figure S1.

In humans, plasma concentrations are approximately proportional to the dose after oral administration of ivermectin.^20^, ^21^ Oral administration of a high single dose of 30 mg ivermectin tablets following a high‐fat meal resulted in an approximate 2.5‐fold increase in bioavailability relative to administration in the fasted state.^20^

After a single dose, the mean values for AUC and C_max_ after administration of 12 mg of ivermectin in ethanol solution were approximately twice as high as after administration of ivermectin tablets 12 mg or ivermectin capsules 12 mg, suggesting that the absorption rate depends significantly on the dosage form. T_max_ values were comparable. Plasma samples were determined by reverse‐phase HPLC with fluorescence detection, with an LLOQ of 0.2 ng/mL.^22^

AUC values from the time point 0 to 72 h (AUC_0‐72h_) of ivermectin 12 mg solution were measured with 1291 ng/mL h.^23^ Extrapolation of these data from the time point 0 to infinity (AUC_0–∞_) resulted in 1473 ng/mL h.^22^ C_max_ values were reported as 82.9 and 81 ng/mL.^22^, ^23^ If these data are extrapolated from a 12 to a 14 mg single dose ivermectin oral solution, pharmacokinetic results are comparable to F004 14 mg single dose values. C_max_ values are determined as 96.6 ng/mL following F004. Extrapolated C_max_ values of ivermectin 14 mg oral solution are 97.7 ng/mL and are thus quite similar. The same applies to T_max_, with 4.0 h measured for ivermectin oral solution and 4.4 h after F004 application. The mean T_max_ value of 4.4 h for F004 indicates a strong mucoadhesive effect of the galenic formulation, besides a systemic effect.

AUC_0–96h_ values of F004 are determined as 1701 ng/mL h, and AUC_0–72h_ values extrapolated to ivermectin 14 mg oral solution are 1506 ng/mL h. AUC values after F004 nasal spray application are about 13% higher (112.9%) compared to the intake of ivermectin oral solution. From these data, it can be concluded that the relative bioavailability after F004 nasal spray application is equivalent to the administration of an oral ivermectin solution.

The small difference could be due to the fact that (a) AUC was determined over a time period of 96 h following F004 but for only 72 h, that is, 24 h less, after ivermectin oral solution, (b) the analytical methods are different to some extent as the data reported on ivermectin B1a concentrations after ivermectin tablets, capsules or oral solution were analyzed with HPLC with fluorescence detection and LLOQ of 1.0 or 1.25 ng/mL^23^ whereas ivermectin B1a concentrations of F004 nasal spray were measured with LC‐MS/MS and LLOQ of 0.250 ng/mL, (c) ivermectin concentrations were only determined in 14 plasma samples per subject after ivermectin oral solution, tablets, or capsules^23^ compared to 16 samples per subject following F004.

Differences in the technical methods and number of plasma samples to analyze ivermectin concentrations may also explain the different half‐life following F004 nasal spray (59.9 ± 20.1 h, 2.5 days) compared to ivermectin tablets (12 or 18 h) as stated in the European summary of product characteristics or in a food interaction clinical study.^20^

Half‐lives of 51 ± 24 h were determined in a bioequivalence study performed in 36 fasted healthy subjects receiving a single dose of ivermectin 9 mg tablet of two different formulations. Twenty‐four samples were analyzed over a time period of 72 h, the analytical method was not reported.^24^ A fixed single dose of ivermectin tablets 18 and 36 mg was administered to 57 fasted healthy subjects in another comparative bioequivalence study. Twenty‐nine plasma samples per subject were withdrawn over a time period of 168 h (7 days). The sum of ivermectin B1a and ivermectin B1b plasma concentrations was analyzed using HPLC/MS/MS (LLOQ = 0.4 ng/mL). Mean half‐lives were determined as 66.85 h (2.8 days) to 100.78 h (4.2 days).^21^ After a single oral dose of ivermectin 18 mg tablets, the mean half‐life of ivermectin B1a was determined as 51 h by LC‐MS/MS. Twenty‐one plasma samples per subject from 70 subjects were analyzed over a time period of 72 h in this clinical trial.^25^ Also, the data on ivermectin solution, tablets, and capsules suggest a longer half‐life.^22^

These half‐life data reported after ivermectin oral administration confirm the ones evaluated after F004 nasal spray application. The long half‐life of up to 100 h is assessed as a result of the high lipid solubility of ivermectin and the associated wide distribution of ivermectin in the body.^21^, ^26^ Ivermectin was detected in all tissue samples removed from various patients infected with *Onchocerca volvulus*. Fat showed the highest concentrations determined by radioimmunoassay after intake of a single oral dose of ivermectin 150 mg/kg bodyweight.^27^

It can be assumed that ivermectin diffuses from adipose tissue back into plasma over a period of several days after treatment termination. This explains why 96% of ivermectin concentrations are still measurable 12 h after the last of a total of 15 F004 nasal spray doses administered over a period of 5 days.

## Conclusion

The safety and tolerability profile of F004 nasal spray was considered high after single and multiple applications, as assessed by the investigator, the subjects, and the ENT specialist. The number of subjects reporting AE and the number of recorded AE decreased toward the middle or end of the 10‐day study period, although the nasal spray continued to be used three times daily. Nasal examination performed by an ENT specialist showed stable patterns of nasal mucosal grading, mucosal bleeding, and crusting of the mucosa.

F004 nasal spray could be an ideal medication to treat patients with COVID‐19 or to prevent the disease. F004 acts directly at the site of entry and replication of the SARS‐CoV‐2 virus and could thus prevent further spread of the virus to the lungs and other organs and act systemically to stop further virus replication in already infected organs, which needs to be investigated through clinical trials in the near future.

## Conflicts of Interest

Stefan Wissel is the owner of HWI Pharma Services GmbH. Philipp Wissel and Felix Häberlein were employees of HWI Pharma Services GmbH. Hilde Riethmüller‐Winzen worked as a consultant on clinical issues for HWI Pharma Services GmbH. Matthias Rischer and Hanns Häberlein worked as consultants on preclinical issues for HWI Pharma Services GmbH.

## Funding

The study was conducted at Nova‐Clin Medical Research Center S.R.L. (Timisoara, Romania) and ACC GmbH (Leidersbach, Germany) and funded by HWI Pharma Services GmbH (Rülzheim, Germany).

## Supporting information



Supporting information

Supporting information

Supporting information

## Data Availability

Data available on request due to privacy/ethical restrictions. The data that support the findings of this study are available on request from the corresponding author. The data are not publicly available due to privacy or ethical restrictions.
