# Supplementary material for: Tolerability, Safety, and Pharmacokinetics of Ivermectin After Nasal Application in Healthy Adult Subjects
Source: J Clin Pharmacol. 2025 Dec 17;66(1):e70137. doi: 10.1002/jcph.70137 (PMC12710153; doi:10.1002/jcph.70137)
Supplement: Supplementary file 1 — Supporting information [file JCPH-66-0-s001.pptx]

## Slide 1
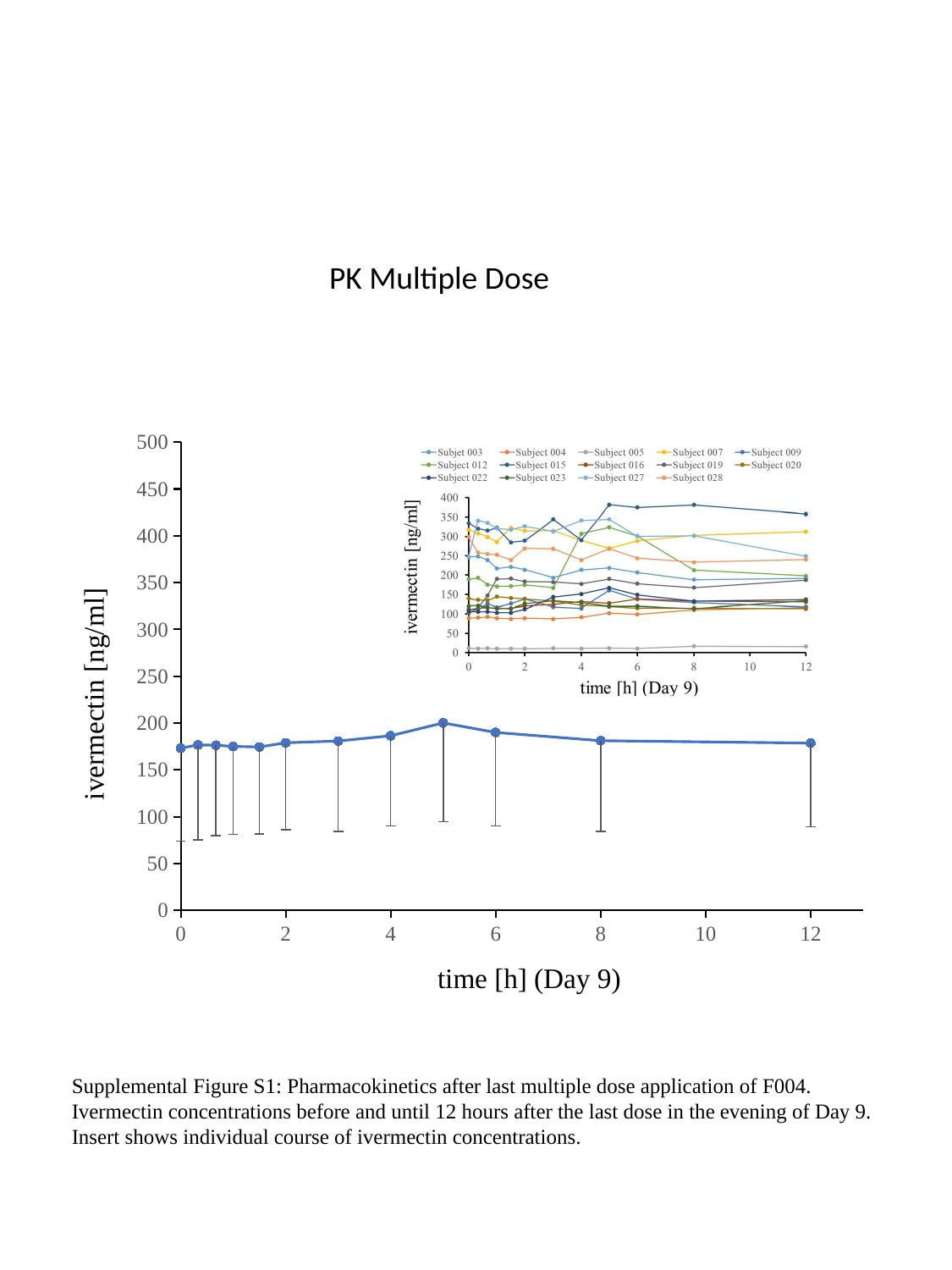

PK Multiple Dose
### Chart
| Category | |
|---|---|
ivermectin [ng/ml]
time [h] (Day 9)
Supplemental Figure S1: Pharmacokinetics after last multiple dose application of F004. Ivermectin concentrations before and until 12 hours after the last dose in the evening of Day 9. Insert shows individual course of ivermectin concentrations.
