# Supplementary material for: Tolerability, Safety, and Pharmacokinetics of Ivermectin After Nasal Application in Healthy Adult Subjects
Source: J Clin Pharmacol. 2025 Dec 17;66(1):e70137. doi: 10.1002/jcph.70137 (PMC12710153; doi:10.1002/jcph.70137)
Supplement: Supplementary file 2 — Supporting information [file JCPH-66-0-s002.docx]

Supplemental Table S1: Demographic data and homogeneity analysis of age and sex proved by Pearson´s Chi-squares test^1^ and linear model ANOVA^2^

|  | **Demographic data** | | | | | | | | | |
| --- | --- | --- | --- | --- | --- | --- | --- | --- | --- | --- |
|  | **total** | | | | | | **Ivermectin suspension 5% (IVM) vs F004 placebo (placebo)** | | | |
|  | **Age [years]** | **Sex: male (m) and female (f)** | **Race (Caucasian)** | **Body mass index (BMI) [kg/m^2^]** | **Height [cm]** | **Weight [kg]** | **Age [years]** | | **Sex: male (m) and female (f)** | |
|  |  |  |  |  |  |  | IVM | Placebo | IVM | Placebo |
| Mean | 33.5 |  |  | 25.7 | 170.9 | 75.4 | 33.6 | 33.4 |  |  |
| SD | 10.7 |  |  | 3.4 | 10.1 | 13.5 | 11.3 | 10.5 |  |  |
| Minium | 18 |  |  | 19 | 150 | 43 | 18 | 19 |  |  |
| Maximum | 58 |  |  | 30 | 188 | 104 | 58 | 51 |  |  |
| Number of subjects | 28 | 13 (m), 15 (f) | 28 | 28 | 28 | 28 | 14 | 14 | 9 (m), 5 (f) | 6 (m), 8 (f) |
| p-value |  |  |  |  |  |  | 0.959^2^ | | 0.256^1^ | |
